# Comparative Study of the Photocatalytic Hydrogen Evolution over Cd_1−x_Mn_x_S and CdS-β-Mn_3_O_4_-MnOOH Photocatalysts under Visible Light

**DOI:** 10.3390/nano11020355

**Published:** 2021-02-01

**Authors:** Ksenia O. Potapenko, Anna Yu. Kurenkova, Andrey V. Bukhtiyarov, Evgeny Yu. Gerasimov, Svetlana V. Cherepanova, Ekaterina A. Kozlova

**Affiliations:** Federal Research Center Boreskov Institute of Catalysis SB RAS, Lavrentieva Ave., 5, 630090 Novosibirsk, Russia; potapenko@catalysis.ru (K.O.P.); kurenkova@catalysis.ru (A.Y.K.); avb@catalysis.ru (A.V.B.); gerasimov@catalysis.ru (E.Y.G.); svch@catalysis.ru (S.V.C.)

**Keywords:** solid solutions, Cd_1−x_Mn_x_S, photocatalysis, hydrogen production, visible light

## Abstract

A series of solid solutions of cadmium and manganese sulfides, Cd_1−x_Mn_x_S (x = 0–0.35), and composite photocatalysts, CdS-β-Mn_3_O_4_-MnOOH, were synthesized by precipitation with sodium sulfide from soluble cadmium and manganese salts with further hydrothermal treatment at 120 °C. The obtained photocatalysts were studied by the X-ray diffraction method (XRD), UV-vis diffuse reflectance spectroscopy, transmission electron microscopy (TEM), X-ray photoelectron spectroscopy (XPS), and N_2_ low temperature adsorption. The photocatalysts were tested in hydrogen production using a Na_2_S/Na_2_SO_3_ aqueous solution under visible light (λ = 450 nm). It was shown for the first time that both kinds of photocatalysts possess high activity in hydrogen evolution under visible light. The solid solution Cd_0.65_Mn_0.35_S has an enhanced photocatalytic activity due to its valence and conduction band position tuning, whereas the CdS-β-Mn_3_O_4_-MnOOH (40–60 at% Mn) samples were active due to ternary heterojunction formation. Further, the composite CdS-β-Mn_3_O_4_-MnOOH photocatalyst had much higher stability in comparison to the Cd_0.65_Mn_0.35_S solid solution. The highest activity was 600 mmol g^−1^ h^−1^, and apparent quantum efficiency of 2.9% (λ = 450 nm) was possessed by the sample of CdS-β-Mn_3_O_4_-MnOOH (40 at% Mn).

## 1. Introduction

Due to the annual growth of world energy consumption and the depletion of fossil fuels, there is a need to develop new types of renewable energy sources. Solar energy has attracted special attention [1,2], as the direct conversion of solar energy into the energy of chemical bonds looks very promising. One of the most effective ways to accomplish the conversion is to reproduce the functions of all types of photosynthesis by creating photocatalytic systems [3]. The ultimate goal in this process is the photocatalytic production of hydrogen [4,5,6]. The unique properties of hydrogen make the element a universal and environmentally friendly chemical energy carrier [7]. From this point of view, the development of new efficient technologies for hydrogen production through the creation of photocatalytic systems that reproduce the functions of photosynthesis is relevant for solving the problems of hydrogen energy [8,9]. Photocatalytic hydrogen evolution is one of the most promising techniques of solar energy storage and conversion [10,11]. The main factor hindering the practical use of photocatalytic processes for hydrogen production is the lack of effective and stable heterogeneous photocatalysts functioning under visible light, which makes up about 43% of the solar spectrum [12].

Cadmium sulfide is one of the first semiconductor photocatalysts to be employed in hydrogen production from aqueous solutions containing organic and inorganic electron donors [13]. Cadmium sulfide is characterized by a band gap of 2.4 eV, which corresponds to the energy of a photon with a wavelength of 520 nm [14]. The positions of the valence and conduction bands of CdS are suitable for the photocatalytic formation of hydrogen under visible light [8,15]. However, the sulfide ion in solid sulfide CdS is prone to oxidize via photogenerated holes and cause photocorrosion of the catalyst [16]. The addition of semiconductors with a large band gap to CdS, such as ZnS or MnS, makes it possible to vary the width of the band gap, the positions of the valence, and the conduction bands in the sample obtained, thereby enhancing the photocatalytic activity of cadmium sulfide [11,12,13,14,15,16,17,18]. At the same time, photocatalysts based on solid solutions of cadmium and zinc sulfides have been studied much more extensively than photocatalysts based on cadmium and manganese sulfides [3,8,16].

The formation of a solid solution requires similarities in the crystal structures and relatively insignificant differences in the radii of the metal cations [8]. Thus, the radius of Mn^2+^ (0.46 Å) is smaller than that of Cd^2+^ (0.97 Å), so cation Mn^2+^ can be incorporated into the CdS lattice with the formation of a solid solution of Cd_1−x_Mn_x_S [17]. Electronegative Cd (1.69) and electronegative Mn (1.55, Pauling’s scale) are also close to each other, which contributes to the formation of composite materials based on CdS and MnS [12]. In this field of study, the formation of Cd_1−x_Mn_x_S solid solutions is considered a promising approach for the synthesis of photocatalysts for hydrogen production. For example, in 2010, Masato Machida et al. [18] synthesized Cd_1−x_Mn_x_S photocatalysts for the first time using the hydrothermal method and showed that this system is quite effective in photocatalytic hydrogen production compared to pure CdS and MnS. At the moment, several articles have been published on the use of the photocatalysts based on a Cd_1−x_Mn_x_S solid solution as an effective catalyst for hydrogen evolution [19,20,21,22,23]. At the same time, expensive organosulfur reagents, such as L-cysteine [17,20,23] and thioacetamide [21,22,23], are commonly used as precursors of sulfur, which is disadvantageous from an economic point of view. A solid solution was synthesized using sodium sulfide in only one publication, and the molar fraction of manganese did not exceed 5% [24].

In addition to the synthesis of solid solutions, the activity of sulfide photocatalysts can be increased through the development of composite systems consisting of sulfide and transition metal oxide/hydroxide. Our recent work showed the high activity of the composite photocatalysts Cd_1−x_Zn_x_S/ZnO and Cd_1−x_Zn_x_S/Zn(OH)_2_ in the production of hydrogen under visible light [25,26]. At the same time, a work considering the prerequisites for the potentially high photocatalytic activity of CdS-Mn_3_O_4_ composite photocatalysts was also recently published [27]. To the best of our knowledge, no comparison has been made between CdS-Mn_3_O_4_ and Cd_1−x_Mn_x_S photocatalysts.

In the present work, we propose for two kinds of photocatalysts, solid solutions of cadmium and manganese sulfides Cd_1−x_Mn_x_S (x = 0–0.35) and composites of CdS-β-Mn_3_O_4_-MnOOH, without any deposited cocatalysts for photocatalytic hydrogen evolution under visible light (λ = 450 nm). A distinctive feature of this synthesis is the use of inorganic sodium sulfide for the precipitation of sulfides. We showed that the peculiarities of this synthesis strongly affect the structure of the solid solution, with the formation of either solid solutions or composites occurring based on solid solutions and different manganese oxides. Both types of photocatalysts showed approximately the same maximum activity at a level of 600 μmol g^−1^ h^−1^, while the stability of the composite photocatalysts was significantly higher. The comparison of these kinds of photocatalysts was carried out for the first time.

## 2. Experimental Section

### 2.1. Photocatalyst Synthesis

The following reagents were used in the synthesis of catalysts: Mn(NO_3_)_2_·4H_2_O (Sigma-Aldrich, USA, 97.0%), CdCl_2_·2.5H_2_O (Vekton, Russia, 98%), Na_2_SO_3_·7H_2_O (Reachim, Russia, 99%) NaOH (Sigma-Aldrich, USA, 98%), and Na_2_S (Biochem Chemopharma, France, 60%).

In the course of this work, two series of photocatalyst samples were synthesized. The synthesis of the Mnx(NaOH) (x = 0; 0.05; 0.1; 0.2; 0.4; 0.6; 0.8; 1.0) series was carried out as follows. The synthesis technique included forming a mixture of hydroxides from salts of the corresponding metals, followed by redeposition with sodium sulfide.

(1) $$\left(1-x\right){\mathrm{CdCl}}_{2}+{\mathrm{xMnCl}}_{2}+2\mathrm{NaOH}\overset{}{↔}\left(1-x\right)\mathrm{Cd}{\left(\mathrm{OH}\right)}_{2}+\mathrm{xMn}{\left(\mathrm{OH}\right)}_{2}+2\mathrm{NaCl},$$

(2) $$\left(1-x\right)\mathrm{Cd}{\left(\mathrm{OH}\right)}_{2}+\mathrm{xMn}{\left(\mathrm{OH}\right)}_{2}+{\mathrm{Na}}_{2}S\overset{}{↔}{\mathrm{Cd}}_{1-x}{\mathrm{Mn}}_{x}S+2\mathrm{NaOH},$$

The suspension was first prepared according to the technique described in detail previously [28]. Then, the precipitates were washed, suspended in 50 mL of distilled water, and placed in a Teflon beaker of an obturator-type autoclave. Hydrothermal treatment was carried out at a temperature of 120 °C for 24 h. Then, the precipitate was centrifuged, washed with distilled water four times, and dried at a temperature of 70 °C for 4 h. These samples are referred to as Mnx(NaOH)—e.g., Mn0.2(NaOH).

The series of **Mnx** was prepared in a similar way, excluding the stage of hydroxide formation. Thus, the primary suspension was obtained according to the reaction.

(3) $$\left(1-x\right){\mathrm{CdCl}}_{2}+{\mathrm{xMnCl}}_{2}+{\mathrm{Na}}_{2}S\overset{}{↔}{\mathrm{Cd}}_{1-x}{\mathrm{Mn}}_{x}S+2\mathrm{NaCl},$$

Then, the residues were treated in an autoclave (120 °C, 24 h), washed, and dried. These samples are referred to as **Mn0.0, Mn0.05**, etc., where the number denotes the mole fraction of manganese embedded in the synthesized sample. For example, Mn0.05 is the sample of Cd_0.95_Mn_0.05_S.

### 2.2. Catalyst Characterization

The obtained catalysts were characterized by various physicochemical methods, including UV-vis diffuse reflectance spectroscopy, X-ray diffraction (XRD), X-ray photoelectron spectroscopy (XPS), transmission electron microscopy (TEM), and low-temperature nitrogen adsorption. Diffuse reflectance spectra of the synthesized photocatalysts were recorded at room temperature in the range of 450–850 nm with a resolution of 1 nm using a Cary 300 spectrophotometer (Agilent, USA). Agilent polytetrafluoroethylene (USA) was used as a standard.

The phase composition of the synthesized samples was determined by X-ray diffraction (XRD). The XRD patterns were recorded on a Bruker D8 diffractometer (Bruker, Germany) using CuKα radiation. Diffraction patterns were performed in the 2Ѳ range from 20 to 70° with a scanning step of 0.05° and an accumulation time of each point of 10 s. The calculation of the lattice constants, average crystallite sizes, and the molar content of the components were carried out using the TOPAS software. The value of the parameter x in Cd_1−x_Mn_x_S was determined using Vegard’s law, constructing a linear relationship between the lattice constant and the manganese concentration in the studied photocatalysts.

The X-ray photoelectron spectra were recorded on a SPECS spectrometer (SPECS, Germany) using AlKα radiation (hn = 1486.6 eV, 150 W). The features of the photocatalysts’ morphology and surface properties were studied using transmission electron microscopes JEM-2010 (JEOL, Japan) and JEM-2200FS (JEOL, Japan). The textural properties of the photocatalysts were studied by low-temperature nitrogen adsorption (77 K) using an ASAP 2400 apparatus.

### 2.3. Photocatalytic Tests

The photocatalytic activity of the synthesized samples was determined in the reaction of hydrogen evolution under visible light radiation with a wavelength of 450 nm. The reaction setup is described in detail in our previous works [29]. A reaction suspension containing 100 mL of 0.1M Na_2_S/0.1M Na_2_SO_3_ and 50 mg of the photocatalyst was placed in the reactor and sonicated for 15 min. During all experiments, the reactor was preliminarily purged with an inert gas (argon) to remove oxygen. Then the reactor was irradiated with a LED light source (0.33 A, 30 W, 45 mW cm^−^^2^); the reaction suspension was continuously stirred using a magnetic stirrer. For the quantitative determination of the evolved hydrogen, a chromatograph Khromas GKh-1000 (Russia) with a NaX zeolite column was used; argon was used as a carrier gas.

The calculation of the apparent quantum efficiency (AQE) was carried out according to the following formula:

(4) $$\mathrm{AQE}=\frac{N_{e^{-}}}{N_{ph}}=\frac{2\cdot W_{H_{2}}}{N_{ph}}\cdot 100\%,$$

where $W_{H_{2}}$ is the rate of photocatalytic hydrogen evolution (μmol min^−^^1^), $N_{ph}$ is the number of photons from light source per unit time (photon min^−^^1^). It was calculated that $N_{ph}$ = 2.1 10^19^ photon min^−^^1^ or 35 μmol (photon) min^−^^1^. Coefficient 2 in the equation of quantum efficiency takes into account the number of electrons involved in the hydrogen production reaction.

## 3. Results and Discussion

### 3.1. Photocatalyst Characterization

**Figure 1:**
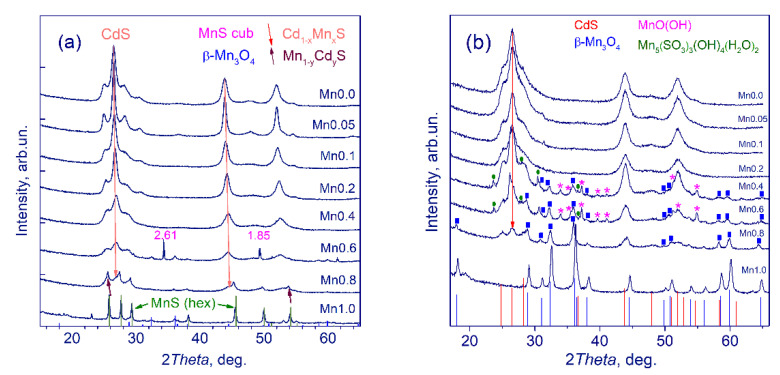
X-ray diffraction (XRD) patterns of the samples **Mnx** (x = 0–1.0) (**a**) and **Mnx(NaOH)** (x = 0–1.0) (**b**).

All photocatalysts were characterized by the XRD technique. Figure 1 shows the XRD patterns of the photocatalysts **Mnx** (x = 0–1.0) (a) and **Mnx(NaOH)** (x = 0–0.8) (b); the structural properties of the samples are shown in Table 1.

The obtained **Mnx** series samples had a highly defective cubic structure with a low particle size. According to the XRD patterns, the best method for the formation of solid solutions is the use of the direct deposition of cadmium and manganese sulfides according to Equation (3) with further hydrothermal treatment. In this case, we observed not only a sequential shift of the sample diffraction peaks towards larger 2Θ angles but also a gradual decrease in the lattice constant with an increase in the molar fraction of Mn (Table 1). This trend is consistent with Vegard’s law and indicates a homogeneous structure. The highest manganese content, 35 at%, was achieved for the photocatalyst **Mn0.4**: a single-phase solid solution Cd_0.65_Mn_0.35_S was formed. Notably, earlier with the use of sodium sulfite as the sulfur precursor, the atomic ratio of manganese did not exceed 5% [24]. For the samples **Mn0.6** and **Mn0.8,** two-phase Cd_0.65_Mn_0.35_S/MnS and Cd_0.65_Mn_0.35_S/Mn_0.92_Cd_0.08_S structures were formed. Note that the Cd_1−x_Mn_x_S crystalline size sharply grew from 7.1 to 13 nm when moving from CdS to Cd_0.95_Mn_0.05_S and then fell monotonously from 13 to 4.3 nm when Mn at% grew from 0.05 to 0.8. The XRD pattern of the **Mn1.0** sample possesses a hexagonal well crystallized MnS structure with a particle size of approximately 37 nm; impurities of β-Mn_3_O_4_ were also observed in XRD pattern of the sample **Mn1.0**. Also, very insignificant amounts of β-Mn_3_O_4_ were identified in the XRD patterns of the samples **Mn0.6** and **Mn0.8**.

Conversely, for the **Mnx(NaOH)** series of samples (x = 0–1.0), the formation of solid solutions practically did not occur; the highest manganese content in Cd_1−x_Mn_x_S was 5 at%. Beginning with *x* = 0.4, the XRD patterns acquired a complex form, where β-Mn_3_O_4_, MnOOH, and even impurities of the Mn_5_(SO_3_)_3_(OH)_4_(H_2_O)_2_ phase appeared. Such complicated **Mnx(NaOH)** (x = 0.4–0.8) phase content was probably caused by the formation of various oxide manganese compounds during the interaction of manganese cations with alkali during synthesis (Equation (1)). When manganese and cadmium chloride interact with alkali (Equation (1)), a white precipitate of cadmium and manganese (II) hydroxides is formed. However, at ambient conditions manganese hydroxide transforms into manganese (III) hydroxide (Equation (5)), also manganese (II/III) oxide is formed in an oxygen-containing atmosphere (Equation (6)).

(5) $$4Mn{\left(OH\right)}_{2}+O_{2}\overset{}{↔}4MnOOH\;+\;2H_{2}O$$

(6) $$3Mn{\left(OH\right)}_{2}+\frac{1}{2}O_{2}\overset{}{↔}Mn_{3}O_{4}\;+\;3H_{2}O$$

When sodium sulfide solution is added (Equation (2)), these compounds do not react, while cadmium hydroxide transforms into much more insoluble cadmium sulfide. It can explain a complicated structure of synthesized samples **Mnx(NaOH)**. The XRD pattern of the **Mn1.0(NaOH)** sample’s single phase β-Mn_3_O_4_ structure had a crystalline size of 19 nm, whereas **Mn0(NaOH)** was shown to possess a cubic CdS with a crystalline size of ca. 6 nm.

**Figure 2:**
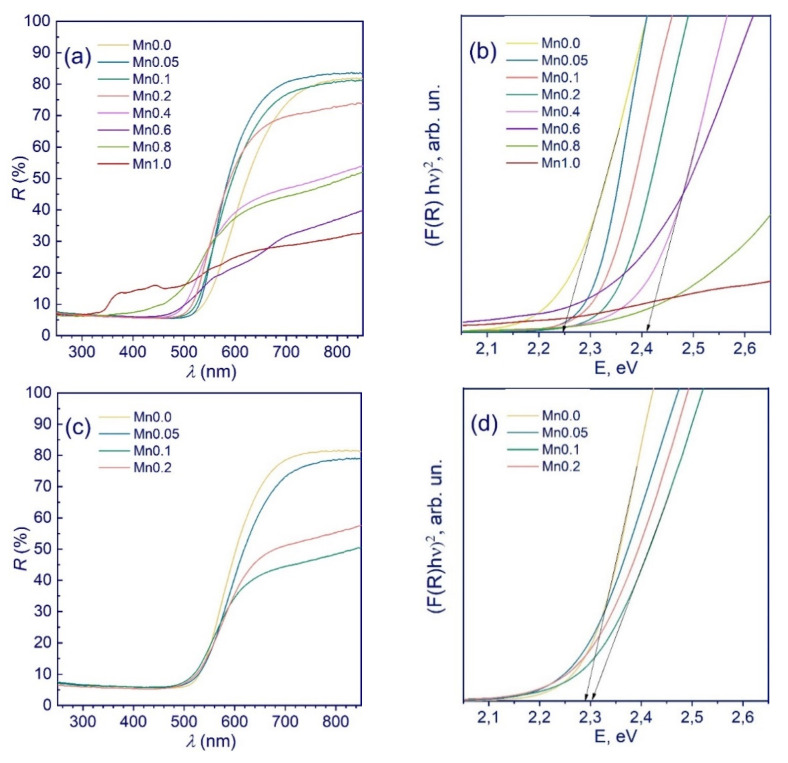
Diffuse reflectance spectra (**a**,**c**) and Tauc’s plot (**b**,**d**) of the samples Mnx (x = 0–1.0) (**a**,**b**) and Mnx(NaOH) (x = 0–0.2) (**c**,**d**).

UV-vis spectroscopy. According to the XRD data, in the case of the **Mnx** (**x = 0**–**1.0**) series, Cd_1−x_Mn_x_S solid solutions without admixtures of manganese oxide compounds were formed. This was also confirmed by the diffuse reflectance spectra shown in Figure 2. Moving from **Mn0.0** to **Mn0.4**, the absorption edges shift monotonically towards shorter wavelengths. With a further increase in the manganese content, the spectra assume a more complex structure, which is explained by the formation of the multiphase samples Cd_0.65_Mn_0.35_S/MnS and Cd_0.65_Mn_0.35_S/Cd_0.18_Mn_0.82_S. Indeed, pure manganese sulfide likely has a very defective structure. According to the curves in Tauc’s coordinates for a direct-gap semiconductor (Figure 2b), an increase in the calculated x in Cd_1−x_Mn_x_S from 0 to 0.35 leads to a monotonic increase in the band gap energy from 2.25 to 2.41 eV. For samples with high manganese content (**Mn0.8**–**Mn1.0**), the calculation of the band gap is quite difficult due to absorption in the entire spectral range.

Figure 2c,d show the UV-vis and Tauc’s plot spectra of the **Mnx(NaOH)** (x = 0–0.2) samples. There is only a very small blueshift in the adsorption edge for the samples with an increase of x. Further, with an increase in the manganese content (x = 0.1–0.2), the photocatalysts exhibit absorption over the entire region of the visible spectrum, which is associated with the presence of impurities of β-Mn_3_O_4_, which are invisible in XRD patterns. The UV-vis spectra of the samples with x = 0.4–1.0 are not shown, because these spectra experience almost complete absorption over the entire spectrum.

**Figure 3:**
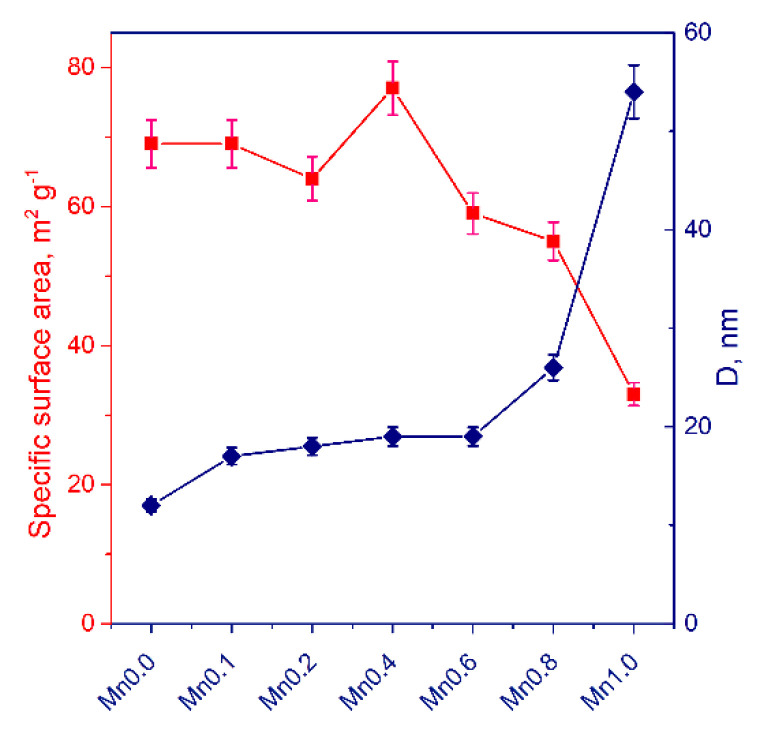
Textural characteristics, specific surface area and pore diameter (D), of the samples Mnx (x = 0–1.0).

Textural properties. Samples of the **Mnx** (x = 0–1.0) series were characterized by low temperature nitrogen adsorption. The properties of these samples are presented in Figure 3. It can be seen that with the incorporation of manganese cations in the cadmium sulfide lattice, the specific surface area first increases and then begins to fall. The decrease in the surface area of the samples **Mnx** (x = 0.6–1.0) with a high proportion of manganese is associated with the formation of manganese sulfide with a large crystalline size as seen from Figure 1a. It was shown that with an increase in x from 0 to 1.0, the average pore diameter increases from 12 to 54 nm, and the pore volume grows from 0.21 to 0.44 cm^3^ g*^−^*^1^. We also studied the texture characteristics of the **Mnx(NaOH)** (x = 0.4–0.8) samples: the specific surface area was 70–80 m^2^ g*^−^*^1^ with pore volume approximately 0.3 cm^3^ g*^−^*^1^. Thus, we can conclude that the presence of the formation stage of hydroxides during synthesis does not have a noticeable effect on the textural properties of the samples but does strongly affect the phase composition.

**Figure 4:**
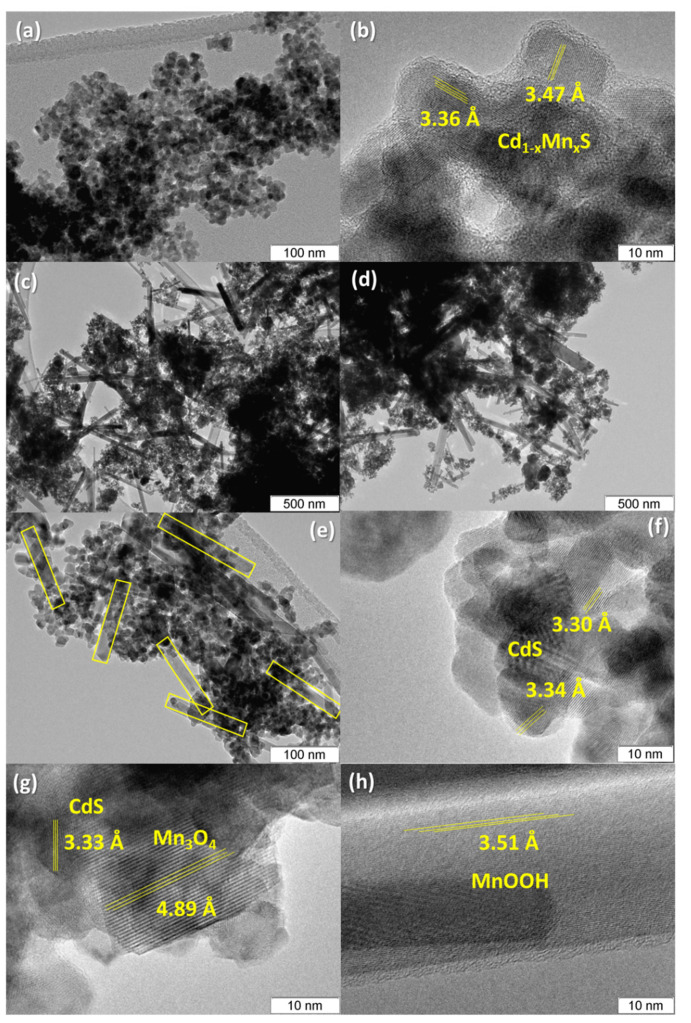
Transmission electron microscopy (TEM) images of Mn0.4 (**a**,**b**) and Mn0.6(NaOH) (**c–h**) samples.

TEM images of the **Mn0.4** and **Mn0.6(NaOH)** samples are shown in Figure 4. For the **Mn0.4** sample (Cd_0.65_Mn_0.35_S), only disordered particles with a size of about 10 nm were observed; these particles are characteristic of Cd_1−x_Mn_x_S solid solutions (Figure 4a,b). The analysis of interplanar spacings showed the presence of a solid solution of manganese and cadmium sulfides Cd_1−x_Mn_x_S.

Thus, the interplanar distances of ca. 0.34 nm shown in Figure 4b corresponds to the interplanar distance d_002_ in the Cd_1−x_Mn_x_S (x ≈ 0.5) structure [30]. Figure 4c–h show the microstructure of the **Mn0.6(NaOH)** sample (Cd_0.98_Mn_0.02_S-β-Mn_3_O_4_-MnOOH). It can be seen that the crystals of the **Mn0.6(NaOH)** photocatalyst have a morphology featuring rod-like structures with lengths of more than 100 nm, which are attributed to particles of manganese oxide or MnOOH, and spherical particles of cadmium sulfide with sizes of around 10 nm (Figure 4c–e). Interplanar spacings equal to 0.33 nm, which correspond to the d_111_ spacing in the cubic CdS structure (PDF # 10-0454), and spacings of 0.49 nm, which correspond to the d_101_ interplanar distance of β-Mn_3_O_4_ (PDF # 24-734), were also observed (Figure 4f–h). The interplanar distance equal to 0.35 nm (Figure 4f) is typical of the d_011_ of MnOOH (PDF # 41-1379). Note that the interplanar distances for CdS and Cd_1−x_Mn_x_S (x = 0.35) are very close to each other. Because the TEM method cannot clearly distinguish between these two structures, we rely on calculations based on XRD patterns.

**Figure 5:**
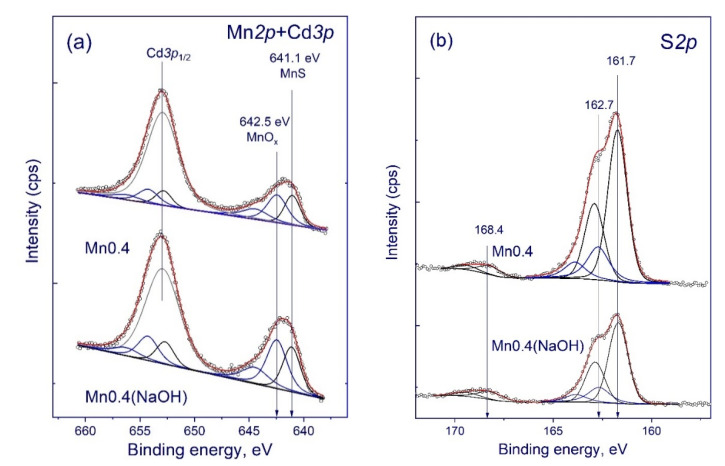
The Mn2p and Cd3p (**a**) and S2p (**b**) X-ray photoelectron spectroscopy (XPS) spectra of the photocatalysts Mn0.4(NaOH) and Mn0.4.

XPS studies. An analysis of the surveyed XPS spectra of samples **Mn0.4(NaOH)** and **Mn0.4** showed the presence of lines characteristic of Cd, S, Mn, and C. To determine the chemical state and ratio of atomic concentrations of the elements on the surfaces of the samples, the spectra of the regions S2p, Cd3d, O1s, Mn2p, and C1s were recorded. The C1s line (284.7 eV) from carbon on the sample surface [31] was used as an internal standard for calibrating the photoelectron peaks. In the Cd3d spectra of both samples, cadmium is present in only one state with a binding energy of 405.4 eV; this value is typical for cadmium in sulfide compositions [32]. In the case of the Mn2p lines of both the **Mn0.4(NaOH)** and **Mn0.4** samples (Figure 5a), the spectrum contains two states of manganese with different values of binding energy. The satellite component (E_b_ = 645 eV, see Figure 5a) indicates that manganese is predominantly in the Mn^2+^ state [33]. The state with binding energy of 642.5 eV can be attributed to the MnO_x_ state, and the binding energy of 641.1 eV is characteristic of manganese in the sulfide composition [30,34].

In the S2p spectra of samples **Mn0.4(NaOH)** and **Mn0.4** (Figure 5b) the sulfur can be in three states with the binding energies of the S2p line: approximately 161.7 eV for sulfur in the sulfide and approximately 168.5 eV for sulfur in the oxidized state in SO_4_^2−^. Further, there is an additional state with a binding energy of 162.7 eV, corresponding to sulfur in the oxysulfide state [35]. Presumably, the formation of oxidized states of sulfur occurs due to contact between the samples and the atmosphere. Table 2 represents the atomic surface ratio of different elements for the **Mn0.4(NaOH)** and **Mn0.4** samples. The main difference between the two series of photocatalysts is their different surface contents of sulfur and oxygen. Photocatalyst **Mn0.4(NaOH),** which contains different manganese oxide species, β-Mn_3_O_4_ and MnOOH, has much more oxygen on its surface than sample **Mn0.4**, which consists of Cd_0.65_Mn_0.35_S. The similar state of manganese in both samples can be explained by the oxidation of highly dispersed manganese under the conditions of the XPS experiment.

Thus, it can be concluded that in the case of the **Mnx** (x = 0–1.0) series, with the direct deposition of sulfides featuring Na_2_S (Equation (3)), solid solutions of Cd_1−x_Mn_x_S (with x up to 0.35), Cd_0.65_Mn_0.35_S-MnS composite photocatalysts, and hexagonal MnS are formed. In the case of the series **Mnx(NaOH) (x = 0**–**1.0)** obtained via the precipitation of sulfides through the formation of hydroxides, CdS (**Mn0(NaOH)**), β-Mn_3_O_4_ (**Mn1.0(NaOH)**), and Cd_1−x_Mn_x_S-β-Mn_3_O_4_-MnOOH (x < 0.05) composite samples were synthesized. Thus, using simple synthetic approaches, it was possible to obtain two fundamentally different series of photocatalysts.

### 3.2. Photocatalytic Activity

**Figure 6:**
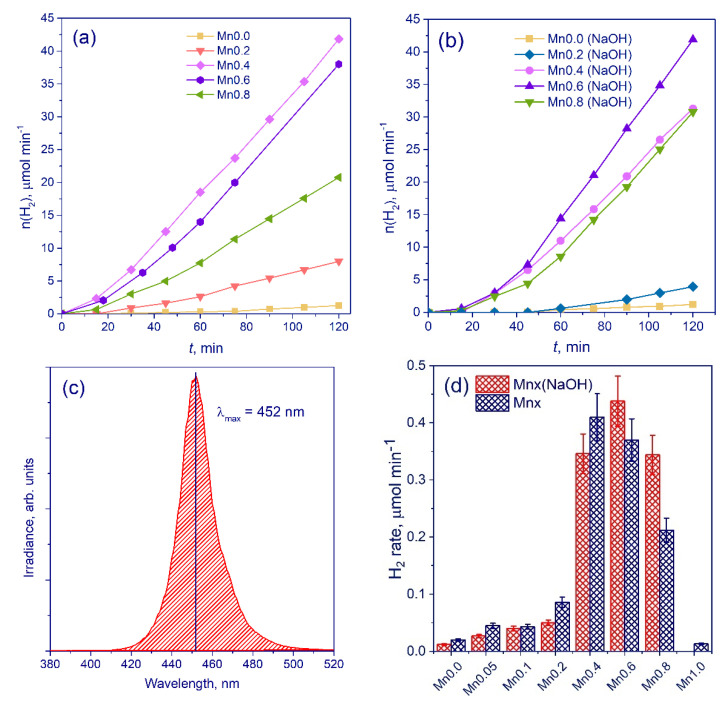
Time plots of hydrogen evolution for series Mnx (**a**) and Mnx(NaOH) (**b**); emission spectrum of 450-LED used as a light source (**c**) and dependence of the rate of photocatalytic hydrogen evolution on the manganese content for the series Mnx (x = 0–1.0) and Mnx(NaOH) (x = 0–1.0) (**d**). Conditions: C_0_(Na_2_S/Na_2_SO_3_) = 0.1 M, C_cat_ = 0.50 g L^−1^, λ = 450 nm.

The activities of the samples of the two series were investigated in the photocatalytic evolution of hydrogen using aqueous solutions containing sodium sulfide and sulfite under visible light irradiation with a wavelength of 450 nm (Figure 6c). It was shown that the rate of the hydrogen evolution was equal to zero without photocatalyst. The time plots of hydrogen evolution were obtained (Figure 6a,b). One can see that for all photocatalysts a constant rate of hydrogen evolution is observed starting from 15 (**Mnx**) and 45 (**Mnx(NaOH)**) minutes of reaction. The reasons for the activation period for the **Mnx(NaOH)** series photocatalysts will be discussed below. The dependences of the rates of photocatalytic hydrogen evolution on manganese content were obtained for all the samples during the study (Figure 6d). The dependences of the reaction rates on the manganese content in the sample had a dome-shaped character with a clearly pronounced maximum. For the series of **Mnx(NaOH)** samples (Figure 6), the formation of a solid solution was not observed, while the high rates were possessed by the photocatalysts **Mn0.4(NaOH)** and **Mn0.6(NaOH)**, consisting of CdS, β-Mn_3_O_4_, and MnOOH. For the sample **Mn0.8(NaOH)** (Cd_0.98_Mn_0.02_S-β-Mn_3_O_4_), a decrease in the rate of photocatalytic hydrogen evolution was observed likely due to absence of MnOOH phase and a very low amount of CdS phase. Earlier it has been shown that Mn_3_O_4_ single phase possesses higher photoluminescence intensity than CdS-β-Mn_3_O_4_ (1:1) composite sample [27]. Thus, composite photocatalysts CdS-β-Mn_3_O_4_ with approximately equal component ratios likely exhibit lower recombination of electron-hole pairs and, accordingly, higher activity than the samples with prevailing manganese oxide content. The single-phase β-Mn_3_O_4_ (**Mn1.0(NaOH)**) was found to possess zero catalytic activity.

On the other hand, for the samples of the **Mnx** series (Figure 6), an increase in activity can be observed when x grows from 0 to 0.4; then, the reaction rate falls. The formation of solid solutions of cadmium and manganese sulfides was observed up to x = 0.4; therefore, an increase in activity with an increase of x in Cd_1−x_Mn_x_S is associated with a change in the band structure of the samples. The samples Cd_0.65_Mn_0.35_S (**Mn0.4**) possessed the highest activity among the photocatalysts of the **Mnx** series. Loss in activity was observed for the composite samples Cd_0.65_Mn_0.35_S/MnS (**Mn0.6**) and Cd_0.65_Mn_0.35_S/Mn_0.92_Cd_0.08_S (**Mn0.8**); extremely low activity of ca. 0.01 mmol H_2_ min^−1^ was observed for the sample MnS (**Mn1.0**). The same tendency was observed when moving from Cd_0.4_Mn_0.6_S to Cd_0.4_Mn_0.6_S/α-MnS [23].

Thus, the highest hydrogen evolution rate was observed for the samples **Mn0.6(NaOH)** (Cd_0.98_Mn_0.02_S-β-Mn_3_O_4_-MnOOH) and **Mn0.4** (Cd_0.65_Mn_0.35_S), whose values were 0.44 and 0.41 μmol min^−1^, or 528 and 492 μmol H_2_ g^−1^ h^−1^, respectively. For both series, an increase in the activity in the production of hydrogen by more than an order of magnitude compared with pristine CdS was achieved. Analysis of the phase composition of the samples and their photocatalytic activity showed that the formation of active photocatalysts for the production of hydrogen requires the formation of a Cd_1−x_Mn_x_S solid solution or CdS- β-Mn_3_O_4_-MnOOH heterostructures. In the first case, the high activity is caused by the formation of a Cd_1−x_Mn_x_S solid solution. It was clearly shown that the formation of Cd_1−x_Mn_x_S solid solutions leads to an increase in the band gap energy and makes the value of the conduction band potential more negative *compared to* NHE [17,30]. According to Table 1, the band gap energies for CdS and Cd_0.65_Mn_0.35_S are equal to 2.2. and 2.4 eV, respectively, whereas the conduction band potentials are equal to around −0.4 and −0.6 V vs. NHE, respectively [17,36,37]. Generally, a more negative potential of the conduction band leads to an easier charge transfer, while still narrow band gap allows efficient visible light absorption; both factors are beneficial for photocatalytic hydrogen production from water [17,38,39]. Further, with more negative conduction band potential, an increase in the reducibility of photogenerated electrons was observed [8].

**Figure 7:**
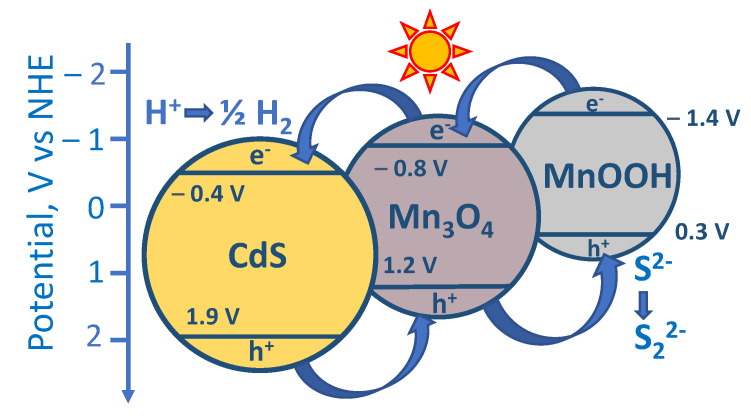
The proposed scheme of the heterojunctions for the photocatalyst CdS-β-Mn_3_O_4_-MnOOH.

It is necessary to consider the reasons for the high photocatalytic activity of the photocatalysts **Mn0.4(NaOH)** and **Mn0.6(NaOH)** consisting of CdS, β-Mn_3_O_4_, and MnOOH. Ternary heterojunctions are likely formed in these composite systems. Thus, it was previously shown that manganese oxide β-Mn_3_O_4_ is a semiconductor with a band gap of around 2.0 eV and a conduction band level position of −0.8 V vs. NHE [40,41,42]. MnOOH has a band energy value equal to 1.7 eV and conduction band potential equal to −1.4 V vs. NHE [42]. Further, it is well known that cadmium sulfide has a band gap of 2.2–2.4 eV and a conduction band at −0.4 eV vs. NHE [8]. Such characteristics imply the absorption of visible light by all three components and the possibility of interfacial heterojunctions between MnOOH, β-Mn_3_O_4_, and CdS (Figure 7). The appearance of triple heterojunctions leads to a significant increase in activity compared to cadmium sulfide. In addition, it is important that the photogenerated holes migrate to MnOOH particles and, accordingly, cannot oxidize the surface of CdS.

**Figure 8:**
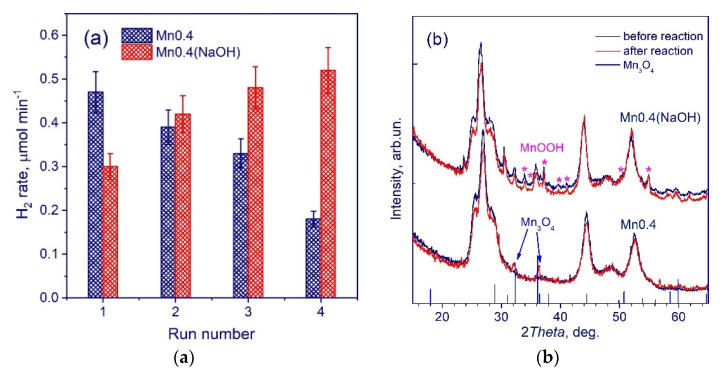
Consecutive photocatalytic runs over the samples Mn0.4(NaOH) and Mn0.4. Conditions: C_0_(Na_2_S/Na_2_SO_3_) = 0.1 M, C_cat_ = 0.50 g L^−1^, λ = 450 nm, 1 run lasted 1.5 h (**a**); XRD patterns of the samples Mn0.4(NaOH) and Mn0.4 before and after the hydrogen evolution (**b**).

We carried out four consecutive photocatalytic runs over the samples of **Mn0.4(NaOH)** (CdS-β-Mn_3_O_4_-MnOOH) and **Mn0.4** (Cd_0.65_Mn_0.35_S) (Figure 8a). A strong deactivation can be observed for the Cd_0.65_Mn_0.35_S photocatalyst, whose rate decreased by a factor of 2.5 in 6 h. Conversely, in the case of the composite CdS-β-Mn_3_O_4_-MnOOH sample, noticeable activation was observed, while the achieved rate of hydrogen production was 0.5 μmol H_2_ min^−1^. The XRD patterns of the samples **Mn0.4(NaOH)** (CdS-β-Mn_3_O_4_-MnOOH) and **Mn0.4** (Cd_0.65_Mn_0.35_S) before and after four photocatalytic runs are shown in Figure 8b. For the **Mn0.4(NaOH)** sample, the XRD patterns are similar, whereas for the **Mn0.4** sample, the single-phase solid solution Cd_0.65_Mn_0.35_S transformed into Cd_1−x_Mn_x_S-β-Mn_3_O_4_, leading to strong deactivation. This means that namely CdS-β-Mn_3_O_4_-MnOOH triple heterostructures are required for efficient hydrogen production. To elucidate the mechanism of activation of the **Mn0.4(NaOH)** sample, an XPS investigation was carried out before and after the hydrogen evolution (Table 2). According to the XPS data, sulfidation of the CdS-β-Mn_3_O_4_-MnOOH photocatalyst surface occurred, leading to an increase in photocatalytic activity. Moreover, Figure 6b represents that the activation period (around 45 min) is observed for the **Mnx(NaOH)** series photocatalysts, especially noticeable for the samples **Mn0.4(NaOH)–Mn0.8(NaOH)**, whereas for the **Mnx** series the period of induction is practically undistinguished. That indicates transformations leading to an increase in the activity. The evolution of hydrogen was carried out using a Na_2_S/Na_2_SO_3_ aqueous solution, and light-induced sulfidation of the surface is quite possible. In addition, according to XPS data (Table 2), in the course of hydrogen production, the proportion of surface sulfide ions increases in comparison with sulfates/sulfites and oxysulfides, which can favorably affect the rate of the process. Thus, the composite photocatalyst CdS-β-Mn_3_O_4_-MnOOH is much more stable than the single-phase Cd_0.35_Mn_0.65_S photocatalyst.

The highest activity was 600 mmol g^−1^ h^−1^, and the maximum value of the apparent quantum efficiency reached 2.9% for the **Mn0.4(NaOH)** sample (λ = 450 nm). These values are not very high in comparison to the recently published data on Cd_1−x_Mn_x_S-based systems [14,15,18,25,38] (Table 3). However, in this work, we used inorganic sodium sulfide for the precipitation of the sulfides. Further, no co-catalysts were deposited on the surfaces of the samples, and the activities were comparable to the data on non-modified Cd_1−x_Mn_x_S.

Thus, the proposed synthetic techniques can synthesize effective materials for photocatalytic hydrogen evolution under visible light irradiation.

## 4. Conclusions

In conclusion, two series of active photocatalysts were synthesized via precipitation with sodium sulfide from soluble cadmium and manganese salts with further hydrothermal treatment at 120 °C. In the case of one-stage precipitation with sodium sulfide, solid Cd_1−x_Mn_x_S (x = 0–0.35) solutions were formed, whereas in the case of two-stage precipitation through the stage of hydroxide formation, composite CdS-β-Mn_3_O_4_-MnOOH photocatalysts were obtained. It was shown for the first time that both kinds of photocatalysts possess roughly the same activity in hydrogen evolution under visible light and tens of times more cadmium sulfide activity. The solid solution Cd_0.65_Mn_0.35_S offered enhanced photocatalytic activity due to its valence and conduction band position tuning, while the CdS-β-Mn_3_O_4_-MnOOH (40–60 at% Mn) samples were active due to the ternary heterojunction formation. Further, the composite CdS-β-Mn_3_O_4_-MnOOH photocatalyst had much greater stability in comparison to the Cd_0.65_Mn_0.35_S solid solution. The highest activity was 600 mmol g^−1^ h^−1^, and the maximum value of the apparent quantum efficiency reached 2.9% for the CdS-β-Mn_3_O_4_-MnOOH (40 at% Mn) sample at λ = 450 nm.

## Figures and Tables

**Table 1:** The phase composition and structural properties, and activities of the synthesized photocatalysts. Conditions: C_0_(Na_2_S/Na_2_SO_3_) = 0.1 M, C_cat_ = 0.50 g L^−1^, λ = 450 nm.

Sample	Cd_1−x_Mn_x_S			Phase Composition	E_g_, eV	W, μmol H_2_ min^−1^	AQE, %
	CS ^1^, nm	a ^2^, Å	x				
**Mnx Series**							
**Mn0.0** (CdS)	7.1	5.84	0.00	CdS	2.25	0.02	0.12
**Mn0.05**	13	5.83	0.03	Cd_0.97_Mn_0.03_S	2.30	0.04	0.22
**Mn0.1**	8.3	5.82	0.07	Cd_0.93_Mn_0.07_S	2.31	0.04	0.22
**Mn0.2**	8.4	5.80	0.20	Cd_0.80_Mn_0.20_S	2.35	0.09	0.52
**Mn0.4**	5.2	5.77	0.35	Cd_0.65_Mn_0.35_S	2.41	0.41	2.34
**Mn0.6**	4.9	5.77	0.35	Cd_0.65_Mn_0.35_S-MnS (cub)	2.38	0.37	2.10
**Mn0.8**	4.3	5.77	0.35	Cd_0.65_Mn_0.35_S-Mn_0.92_Cd_0.08_S	-	0.21	1.20
	10	5.66	0.92				
**Mn1.0**	37	-	1.0	MnS (hex)	-	0.01	0.06
**Mnx(NaOH) Series**							
**Mn0.0** (CdS)	6.1	5.84	0.00	CdS	2.29	0.01	0.06
**Mn0.05**	5.9	5.83	0.05	Cd_0.95_Mn_0.05_S	2.28	0.03	0.16
**Mn0.1**	6.5	5.83	0.04	Cd_0.96_Mn_0.04_S	2.30	0.04	0.22
**Mn0.2**	5.7	5.83	0.03	Cd_0.97_Mn_0.03_S	2.30	0.05	0.28
**Mn0.4**	7.1	5.83	0.02	Cd_0.98_Mn_0.02_S- β-Mn_3_O_4_-MnOOH	-	0.35 0.51 ^3^	2.00 2.91 ^3^
**Mn0.6**	6.4	5.84	0.02	Cd_0.98_Mn_0.02_S- β-Mn_3_O_4_-MnOOH	-	0.44	2.50
**Mn0.8**	7.5	5.83	0.04	Cd_0.96_Mn_0.04_S- β-Mn_3_O_4_	-	0.34	2.00
**Mn1.0**	-	-	-	β-Mn_3_O_4_	-	0	0

^1^ CS—crystalline size; ^2^ a—cubic lattice parameter; ^3^ after 4 photocatalytic runs.

**Table 2:** Surface concentration of elements and compounds calculated from XPS spectra.

Sample	[Mn]/ [Mn+Cd]	[S]/ [Mn+Cd]	[O]/ [Mn+Cd]	[MnS]/ [MnO_x_]	MnS, %	Sulfur Distribution, %		
						S^2−^	Oxy-Sulfide	SO_4_^2−^
**Mn0.4(NaOH)**	0.22	0.47	1.21	0.50	33.3	71.5	19.6	8.9
**Mn0.4(NaOH) ^1^**	0.17	0.77	0.75	0.65	39.4	80.3	12.2	7.5
**Mn0.4**	0.15	0.95	0.39	0.51	34.0	73.3	22.2	4.5

^1^ After photocatalytic test.

**Table 3:** The comparison of the activities of the synthesized samples with recently published data.

№	Photocatalyst	Synthesis	Light Source	Cut-off Filter	Electron Donor	W, μmol h^−1^ g^−1^	Ref.
1	Cd_0.65_Mn_0.35_S	Hydrothermal synthesis; Na_2_S	450 nm LED		Na_2_S/ Na_2_SO_3_	444	Current study
2	CdS-β-Mn_3_O_4_-MnOOH					600	
3	Cd_0.5_Mn_0.5_S	Hydrothermal synthesis; L-Cysteine	300 W Xe lamp	λ > 420 nm	Na_2_S/ Na_2_SO_3_	625	[20]
4	3%MoS_2_/Cd_0.5_Mn_0.5_S					3950	
5	Cu_2−x_S/Cd_0.5_Mn_0.5_S					8090	
6	Cu_2−x_S/Cd_0.5_Mn_0.5_S/3%MoS_2_					13800	
7	Mn_0.05_Cd_0.95_S	Hydrothermal synthesis; thioacetamide	300 W Xe lamp	λ > 420 nm	Na_2_S/ Na_2_SO_3_	1400	[19]
8	NiCoB/Mn_0.05_Cd_0.95_S					10500	
9	Cd_0.5_Mn_0.5_S	Hydrothermal synthesis; L-Cysteine	300 W Xe lamp	λ > 420 nm	Na_2_S/ Na_2_SO_3_	451	[30]
10	1%Pt/Cd_0.5_Mn_0.5_S					2700	
11	0.3%NiS/Cd_0.5_Mn_0.5_S					8390	
12	Cd_0.5_Mn_0.5_S (L-Cysteine)	Hydrothermal synthesis	300 W Xe lamp	λ > 420 nm	lactic acid	444	[23]
13	Cd_0.5_Mn_0.5_S (thioacetamide)					1792	
14	Cd_0.5_Mn_0.5_S (thiourea)					178	
15	Cd_0.5_Mn_0.5_S	Hydrothermal synthesis; L-Cysteine	300 W Xe lamp	λ > 420 nm	Na_2_S/ Na_2_SO_3_	646	[43]
16	0.25% MoS_2_/Cd_0.5_Mn_0.5_S					3940	

## Data Availability

The data presented in this study are available on request from the corresponding author.
